# Reference values for right atrial and right ventricular morphology and function using cardiovascular magnetic resonance imaging

**DOI:** 10.1186/1532-429X-15-S1-E115

**Published:** 2013-01-30

**Authors:** Florian Andre, Cihan Celik, Mohamed A Abdelrazek, Maria Fernanda Braggion Santos, Sebastian A Seitz, Dirk Lossnitzer

**Affiliations:** 1 Department of Cardiology, University of Heidelberg, Heidelberg, Germany; 2 Radiology, Cairo university, Faculty of Medicine, Cairo, Egypt; 3 School of Medicine of Ribeirao Preto University of Sao Paulo, Sao Paulo, Brazil

## Background

The right ventricular (RV) morphology and function are important parameters for the diagnosis of cardiopulmonary diseases and serve as predictors for the long-term outcome. To date there are little data about right heart characteristics due to its complex anatomy. Since cardiac magnetic resonance (CMR) is the gold-standard for the evaluation of left and right cardiac anatomy and function we sought to investigate and provide reference values for the entire right heart acquired in a large population of healthy volunteers.

## Methods

We studied a group 119 healthy volunteers (60 male, 59 female) which consisted of three age groups of nearly equal size (I: 20-34 yrs.,II: 35-49 yrs.,III: ≥50 yrs.). CMR images were acquired on a 1.5 T whole body MRI applying a standard cine SSFP sequence. Right atrial (RA) function and morphology as well as RV sizes were assessed in a 4-chamber view. For the measurement of the RV volumes and function short axis views were used. End-diastolic and end-systolic volumes and diameters (EDV, ESV, EDD, ESD), RV stroke volume and ejection fraction (SV, EF) were measured. As the emptying of the RA is a combination of active and passive mechanisms we tried to quantify the contribution of the contraction by measuring the RA contractive stroke volume (RA CSV) and ejection fraction (RA CEF). Furthermore the RV end-diastolic and end-systolic length (EDL, ESL) and the RA end-diastolic and end-systolic area (EDA, ESA) were quantified. Body surface area (BSA) was calculated using the Mosteller formula. A p-value <0.05 was regarded as statistically significant.

## Results

**Figure 1:**
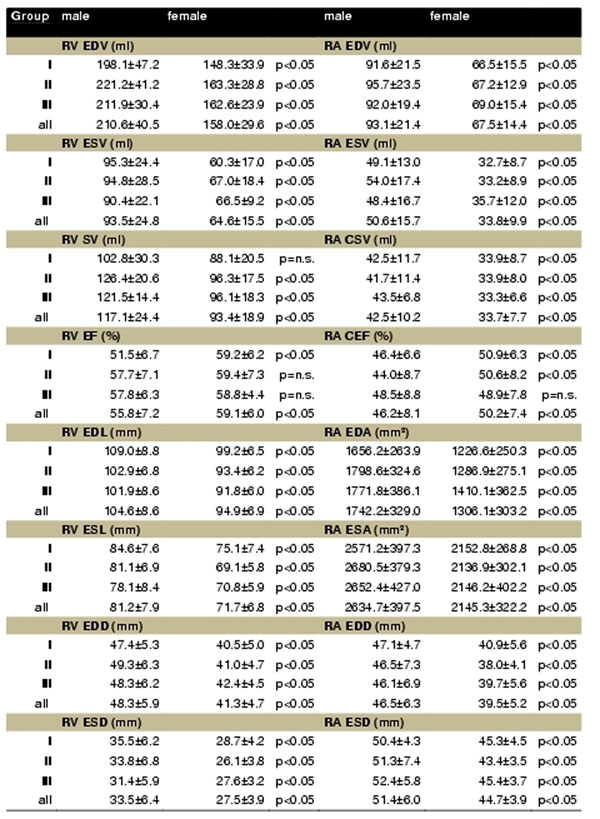
Reference values for RA and RV morphology and function

The reference values are shown in figure 1. The general differences between men and women yielded significance in all parameters. When indexed to BSA the discrepancies between male and female healthy volunteers were still significant regarding EDV, ESV, SV, RA EDA and RA ESA. Regarding the diameters and lengths indexed to BSA only the gender difference of the RV ESD yielded significance.

## Conclusions

In this study we provide age- and gender specific reference values for the right heart morphology and function in a large group of healthy volunteers applying a standard clinical SSFP sequence. These values may contribute to define a reference frame to detect pathological alterations. Yet, further studies are needed confirm our results and to define cut-off values applicable for clinical routine.

## Funding

none

